# Supplementary figures and images for: Deep learning approach based on superpixel segmentation assisted labeling for automatic pressure ulcer diagnosis
Source: PLoS One. 2022 Feb 17;17(2):e0264139. doi: 10.1371/journal.pone.0264139 (PMC8853507; doi:10.1371/journal.pone.0264139)

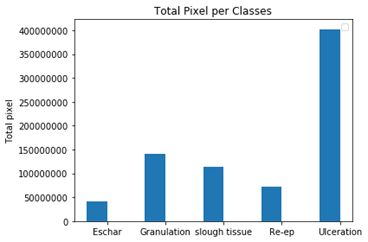

Supplement: S1 Fig — The pixels of ulceration are slightly more than the sum of pixels of granulation, slough, and eschar. (TIF) [file pone.0264139.s001.tif]

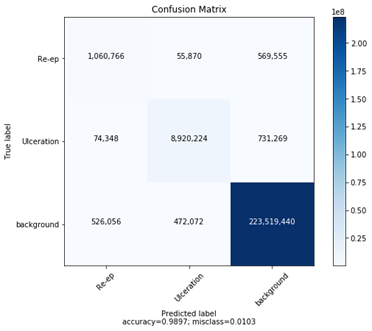

Supplement: S2 Fig — The matrix showed the results when re-ep was trained with ulceration. (TIF) [file pone.0264139.s002.tif]

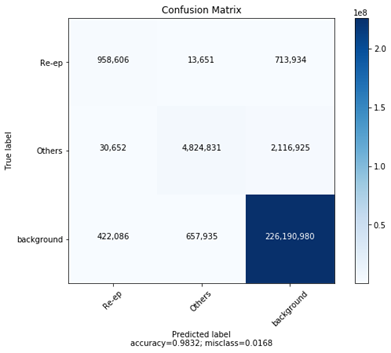

Supplement: S3 Fig — The matrix showed the results when re-ep was trained with granulation, eschar and slough (sum up as others). (TIF) [file pone.0264139.s003.tif]

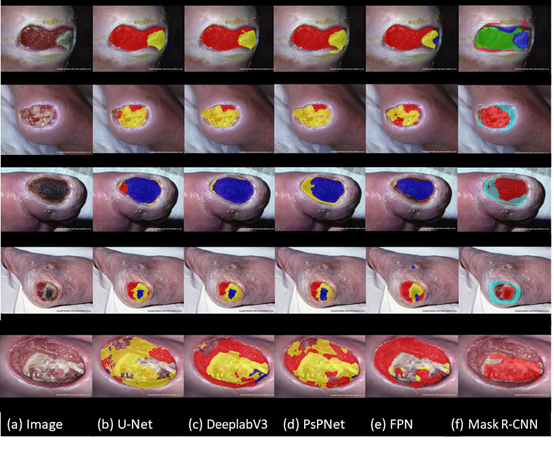

Supplement: S4 Fig — (TIF) [file pone.0264139.s004.tif]

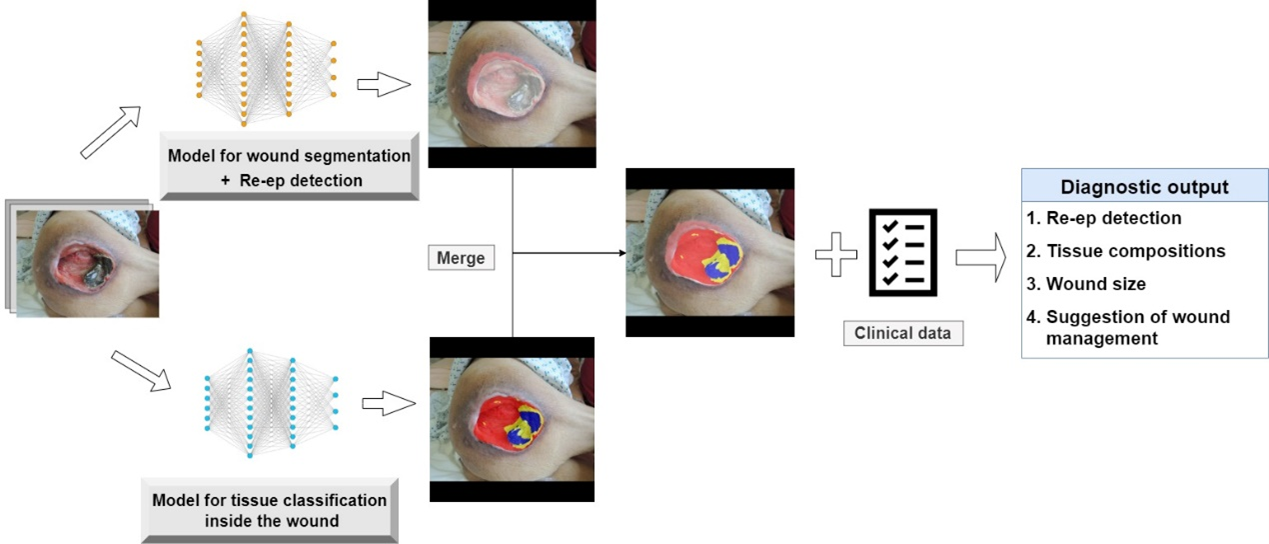

Supplement: S5 Fig — (TIF) [file pone.0264139.s005.tif]

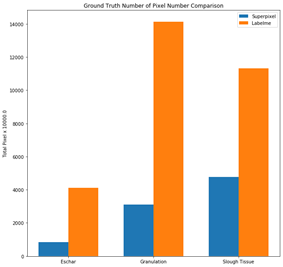

Supplement: S6 Fig — The total number of pixels of different tissues in the labelme dataset (orange bar) and superpixel dataset (blue bar). (TIF) [file pone.0264139.s006.tif]
