# Supplementary material for: Deep learning approach based on superpixel segmentation assisted labeling for automatic pressure ulcer diagnosis
Source: PLoS One. 2022 Feb 17;17(2):e0264139. doi: 10.1371/journal.pone.0264139 (PMC8853507; doi:10.1371/journal.pone.0264139)
Supplement: Attachment — Submitted filename: Response to Reviewers.docx [file pone.0264139.s009.docx]

**Dear Majeed**

We thank you and reviewers for the insightful comments and general enthusiasm to our work. We have incorporated their critiques in the revised manuscript, which we believe has strengthened the manuscript considerably. Our responses to the individual comments are given below.

1. Please ensure that your manuscript meets PLOS ONE's style requirements.

***Answer:*** We apologize for not following the format of this great journal. We had made major revisions to the manuscript body, including all tables, figures, and supporting materials. We are sorry for the inconvenience.

2. We note that your paper includes detailed descriptions of individual patients/participants.

***Answer：***This study was approved by the research ethics review committee of the Far Eastern Memorial Hospital (No. 109145-E). All images of wounds were downloaded from EMR and randomly assigned numbers before labeling. All images do not include any part of the body that can be used to identify patients, such as faces, birthmarks, tattoos, and special dressings. Our manuscript does not include detailed descriptions of individual participants. We are unable to trace the ID of patients from processed images of wounds. The datasets of de-identification images will be deleted in a given period and DL models can be preserved.

3. Thank you for stating the following in the Acknowledgments Section of your manuscript.

***Answer:*** We apologize for not reading the guideline thoroughly again. We would like to move “"This work was supported by the Innovation Project of the Far Eastern Memorial Hospital (Grant No. PI20200002)” to the Funding Statement section.

4. We note that you have stated that you will provide repository information for your data at acceptance.

***Answer:*** We apologize for the misunderstanding. We considered the repository information to be figures and supporting materials in the manuscript instead of the labeled dataset. According to the statement of our research ethics review committee, the images downloaded from EMR could only be used for research of our own hospital, but not as an open dataset. We believe that there is a consensus of all hospitals around the world, which is unable to assess the images of wounds publicly.

Currently, there are around 200 articles of ML in wounds. In every articles, ML models were trained from individual datasets. There is no open dataset of images of wounds except the *Medetec* wound database. We included the precious images from *Medetec* to our testing set and presented the results in the figures of manuscript.

To solve this common problem, we have tried federated learning, a hierarchy back-end structure of ML, to combine weights from many local models without sharing the individual datasets. We hope our work would complete recently and submit to your great journal soon!

5. In your Data Availability statement, you have not specified where the minimal data set underlying the results described in your manuscript can be found.

***Answer:*** We do not know the minimal data required to achieve the results. However, our preliminary study had used dataset with 755 images. The F1 score were 0.6-0.5 and recall were 0.75-0.82 by U-Net and Mask R-CNN. In the literature review, Ortiz et al. (DOI: 10.1186/s12938-016-0298-3) used 51 images, Veredas et al. (DOI: 10.1109/TMI.2009.2033595) used 113 images, Mesa et al. (DOI: 10.1109/HIS.2008.33) used 113 images, and Kosmopoulos et al. (DOI: 10.1109/EMB.2007.901786) used 85 images with various outcomes. We applied more than 2800 images of pressure ulcers in both datasets. Speaking to deep learning, data are never too many.

We feel sorry that we are unable to upload our labeled dataset by the regulation of our research ethics review committee. Instead, we are keen to share the images of *Medetec* wound database, which are labeled by our proposed methods (boundary-based and region-based) to verify our study. The DOIs are ([**https://doi.org/10.6084/m9.figshare.17206904.v1**](https://doi.org/10.6084/m9.figshare.17206904.v1) and [**https://doi.org/10.6084/m9.figshare.17206940.v1**](https://doi.org/10.6084/m9.figshare.17206940.v1)) The information were added to our supporting information in the manuscript.

Best Regards and Merry Christmas

Dr. Che Wei, Chang

**Dear reviewer 1**

3.2 Total and breakdown of the data images collected should be mentioned here. Also, were all these images taken with the same parameters such as distance from the wound to the camera, camera angle and such. Obviously, the same wound would look bigger and have more pixels if it is nearer to the camera than if it is further away. This should be clarified.

***Answer*:** Thank you for this critical observation. The images of pressure ulcers were download from our EMR, which were taken by various types of devices, including cellphones, pads and cameras. The conditions of taking pictures (illumination, background, distance, and angle) were also diverse. We had tried using a small number of images being taken under the standard condition to train models as in previous articles. Although these models had good results only in the training dataset, their performance was poor when facing images of other patients. That is unexpected but not surprised. It is important for deep learning models to comprehend information from any condition.

Since the images were downloaded from the EMR of our hospital, we did not know the exact distance and camera angle of every image. However, in our dataset, the average ratio of wound area to the whole picture is around 44%. The distance too far or wound too small were difficult to be labeled and will be excluded by plastic surgeons.

3.3 This section should be more concise. The division between inside and outside ulceration should be explained more directly. I think this can be explained better using either point form format or listing in down in a table.

***Answer*:** We thank the reviewer for your insightful comment. To address this comment, we had condensed the definition and description of different types of tissues into Table 1.

| **Table 1. Tissues of pressure ulcer inside and peri-wound.** | | |
| --- | --- | --- |
| Type | Color | Description |
| Granulation (Inside) | Beefy red to pink | 1. Healthy tissue in wounds  2. Collagen, fibroblast, capillary  3. Indication of wound healing |
| Eschar (Inside) | Black to brown | 1. Desiccate, nonviable tissues from skin, fat, tendon, or muscle  2. Usually clear margin if stable |
| Slough (Inside) | Mostly yellow, could be white, tan, grey or green | 1. Most varied appearance  2. Partially dead or complete necrotic  3. Mix with fibrin, biofilm or bacteria |
| Re-epithelialization  (Peri-wound) | Light pink to purple | 1. Formation of wound bed matrix  2. Migration of keratinocytes  3. Indication of wound healing |

3.4 What were the 4 models used to test the segmentation? And why did the author use 4 different models for this? It is not mentioned in this section. The text in Fig 2 is too small and unreadable.

***Answer*:** We apologize for not providing clarity while discussing the effect of classes combination. This paragraph demonstrated that a model has better sensitivity and DC for re-ep when re-ep training with ulceration than granulation + eschar + slough, although ulceration ≈ granulation + eschar + slough. In aspect of re-ep, the partitions will result four models, but only models with re-ep were used for comparison. Our description was slightly confusing for which we rewrote the paragraph to be clearer.

| Re-ep + ulceration | Granulation + eschar + slough |
| --- | --- |
| Ulceration | Re-ep + granulation + eschar + slough |

3.5 Perhaps the surgeons found it difficult to agree on the regions because they were not specifically defined for them? As in this paper, there was no specific definition on how the different tissues mentioned were defined. Figure 5 is more confusing than it should be. The flow of work should be clearer and any graphics that are not important should be removed.

***Answer:*** As your professional suggestions, we condensed the definition and description of tissues into Table 1. They have specific definitions but still hard to be labeled. Most tissues inside and peri-wound have a various range of colors and texture. It is unlikely to take every piece of tissue on the wounds for biopsy to confirm. Images can only be co-labeled by experienced plastic surgeons as “relative” ground truth. In our initial dataset, there were many excluding images because of no consensus on labeling. Even within the labeled images, there were still pixels difficult to be labeled and left undefined. The relation can be described as Ulceration = (Granulation ∩ Eschar ∩ Slough ∩ Undefined).

We had revised Figure 5 to be more legible. We separated the labeling & training from automatic diagnosis to make new Figure 4 and S5 Figure.

**Fig 4. The workflow of labeling and training**. Boundary-based labeling was used for peri-wound tissue and ulceration. Region-based labeling was applied for inside wound tissues.

**S5 Figure The workflow of automatic diagnosis and the outputs.**

5. Discussion
- the discussion is not focused and does not expand on the findings, and instead introduces new concept and results. There is also quite a lot of information that is repeated from previous sections.

***Answer:*** We appreciate this critique from the reviewer. The first paragraph of discussion was redundant. We had trimmed down to make it more concise. There were two main parts in the discussion. The first part was the expanding of superpixel segmentation from the evolving of this type of ML. After superpixel segmentation processing, we input fewer pixel numbers but achieved better results in a manner of improving inter-rater consistency. In the second part, we tried to explain why different classes combination has an impact on the recall (sensitivity) of a specific class. Re-ep is an important class but has small number of pixels with irregular shape.

6. Overall formatting needs to be rechecked and redone. For example, most paragraphs are not aligned correctly. Same goes for figures (which still have boxes around them) and equations. While the first half of this paper is quite good, the latter half seems rushed and does not reach the quality expected after reading the first half. I hope that the authors can present their work (which seems like a lot of work) in a way that is easier to understand and appreciate.

***Answer:*** We apologize for not following the format of the journal. We are grateful for your general enthusiasm to our work. We had revised all our tables, figures, and supporting materials, including re-drawing some figures to make them legible. The discussion was also revised to be adhesive to the results.

Best Regards and Merry Christmas

Dr. Che Wei, Chang

**Dear reviewer 2**

We apologize for not following the format of the journal. We are grateful for your general enthusiasm to our work.

1. Section 3 - Methods - Naturally the evaluation metrics described in 3.1 should be moved at the end of the methods section.

***Answer:*** We thank the reviewer for this thoughtful comment. We had moved the evaluation metrics before the results of different combination were presented. We apologize for not following the format of this great journal. We also revised all our tables, figures, and supporting materials, including re-drawing some figures to make them clearer.

2. Why was the ResNet101 used as the encoder for all models evaluated? Please substantiate the selection through literature, in the even a sensitivity test was not carried out.

***Answer:*** We are grateful for this insightful comment by the reviewer. ResNets are powerful encoder that can be compatible with any deep learning models and are easy to optimize. Our preliminary studies had tried ResNet50, ResNet101, and ResNet152 with U-Net. The U-Net with ResNet101 had much better performance than with ResNet50. It is interesting that U-Net with ResNet152 only had a marginal advantage than with ResNet101. ResNet152 had more likely to experience overfitting and consumed more computing resource. Based on the previous result, we applied ResNet101 for all deep learning models in our final presentation. We had added descriptions to **Methods- Deep learning models** regarding the choice of an encoder.

Best Regards and Merry Christmas

Dr. Che Wei, Chang
